# *Plasmodium falciparum* Malaria Complicated by Symmetrical Peripheral Gangrene, Bowel Ischemia, Repeated Candidemia, and Bacteraemia

**DOI:** 10.1155/2014/696725

**Published:** 2014-04-09

**Authors:** Emeline Masse, Philippe Hantson

**Affiliations:** ^1^Département des Soins Intensifs, Université Catholique de Louvain, Cliniques Saint-Luc, Avenue Hippocrate 10, 1200 Brussels, Belgium; ^2^Louvain Centre for Toxicology and Applied Pharmacology, Université Catholique de Louvain, Cliniques Saint-Luc, Avenue Hippocrate 10, 1200 Brussels, Belgium; ^3^Department of Intensive Care, Cliniques Saint-Luc, Avenue Hippocrate 10, 1200 Brussels, Belgium

## Abstract

A 63-year-old Caucasian woman developed severe *Plasmodium falciparum* malaria when travelling back from Cameroun. No antimalarial chemoprophylaxis had been observed. The patient was immediately admitted to the intensive care unit after evidence of multiple organ failure (coma, shock, acute respiratory distress syndrome, acute renal failure, etc.). However, initial parasitemia was less than 1%. The patient was managed by intravenous quinine and norepinephrine infusion due to refractory shock. The patient developed as an early complication ischemic lesions of both arms and feet. In addition to laboratory changes consistent with disseminated intravascular coagulation, there was also evidence for a low activity of the von Willebrand factor (VWF) cleaving protease ADAMTS13. Later complications included repeated candidemia and bacteraemia despite appropriate therapy; the origin appeared to be diffuse ischemic injury of the gastrointestinal tract. The patient ultimately recovered, but quadriamputation was necessary to treat symmetrical peripheral gangrene (SPG). In severe *Plasmodium falciparum* malaria, ischemic changes may be due to microvascular obstruction, but, in patients with low parasitemia, other endothelial factors may also be involved as observed in other groups of thrombotic microangiopathies.

## 1. Introduction

Symmetrical peripheral gangrene (SPG) is a possible but still infrequent complication of* Plasmodium falciparum *malaria [1–13]. The mechanism is usually ascribed to the activation of the coagulation pathway and to microvascular obstruction [5]. More recently, the role of ADAMTS13 (a von Willebrand factor (VWF) cleaving protease) activity has also been discussed to explain malaria-related microangiopathy [14]. Early coinfection with bacteria or even fungi may be the consequence of a relative immunosuppression and increased intestinal permeability [15]. We describe herein a severe case illustrating these acute pathophysiological changes.

## 2. Case Report

**Figure 1:**
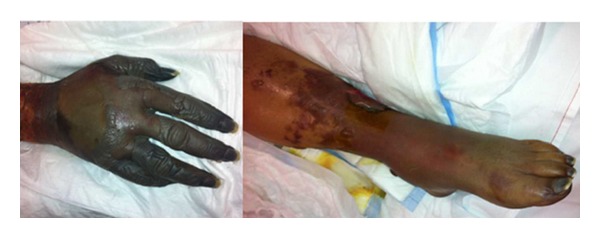
Aspect of the symmetrical peripheral gangrene affecting both arms and legs after 1 month in the ICU.

**Figure 2:**
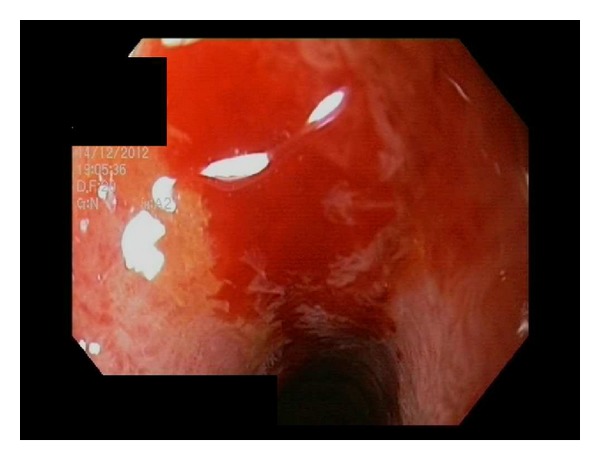
Gastroscopy performed on day 11 and showing diffuse hemorrhagic lesions in the stomach and duodenum, with an ischemic pattern of the mucosa.

**Figure 3:**
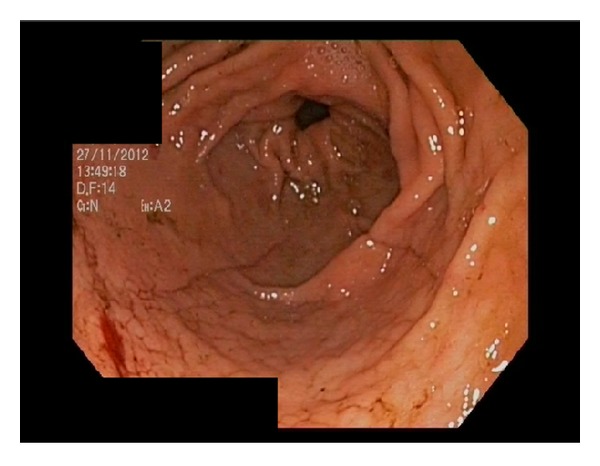
Colonoscopy performed on day 28 and showing diffuse ischemic lesions mainly in the sigmoid colon.

A 63-year-old Caucasian woman without medical history stayed for several weeks in Cameroun, without taking any antimalarial chemoprophylaxis. She developed severe gastrointestinal symptoms and, after three days, due to her suspicion of a malaria attack, she took at once four pills of Coartem (each containing 20 mg artemether and 120 mg lumefantrine). After that, the patient became unable to ingest any food or drug and a transfer to Europe was rapidly decided. She became seriously ill during the flight back. On arrival at the airport, she was found stuporous and hypotensive and was rapidly transferred to the intensive care unit (ICU) of the nearest hospital. The diagnosis of* Plasmodium falciparum* malaria was rapidly made on the blood smear and treatment was started with intravenous quinine and doxycycline (as artesunate was not available). Parasitemia was estimated less than 1%. Admission coagulation tests revealed activated partial thromboplastin time 37 sec, international normalized ratio 1.2, fibrinogen 299 mg/dL, D-dimers >8000 ng/mL (normal, <500), ADAMTS13 activity undetectable (reference range, 50–150%), von Willebrand factor (VWF) antigen 340% (reference range, 50–150%), and platelet count 21,000/mm³. Early complications included refractory hypotension and hypoxemia and neurological worsening. Intubation was required for mechanical ventilation; norepinephrine infusion was progressively increased to the maximal infusion rate of 46 *μ*g/min. An acute left ventricular dysfunction could be demonstrated at echocardiography. Continuous venovenous hemofiltration was initiated for acute renal failure. Due to the evidence of multiple organ failure and despite relatively low parasitemia, exchange transfusion therapy was performed. Only one session of 87 minutes was performed. The patient's theoretical total blood volume was 4407 mL; on the whole, 1409 mL of red blood cells was removed and replaced by 1277 mL of packed red blood cells. Blood coagulation tests remained disturbed, with epistaxis and rectal bleeding. The nadir of platelet count (5,000/mm³) and plasma fibrinogen (66 mg/dL) was noted on day 4, but there was only a mild increase in activated partial thromboplastin time and prothrombin time. The activity of ADAMTS13 slightly increased to 32%; no inhibitor or ADAMTS13 autoantibodies were found. From day 2, blackish discoloration of both hands and feet was noted and by the following days a definite line of demarcation was observed between gangrenous and normal skin (Figure 1). Ultrasound-Doppler examination confirmed the absence of distal perfusion. Plastic surgeon suggested postponing the extensive surgery. Further clinical course was characterized by repeated candidemia (days 4, 7, 9, 10, and 11) and bacteraemia (*Pseudomonas aeruginosa* on day 7 and* Enterococcus faecalis* on days 12, 13, and 18), despite appropriate antifungal and antimicrobial therapy. The abdomen computed tomography (CT) failed to reveal bowel ischemia, but diffuse thickening of the jejunal wall was found. Endoscopy on day 11 disclosed multiple duodenal ulcerations from ischemic origin (Figure 2); colonoscopy performed on day 28 was also consistent with ischemic injury disseminated in the sigmoid colon (Figure 3). Norepinephrine infusion had been definitely stopped on day 14. The patient regained consciousness progressively from day 22 and extubation was possible on day 32.

The decision to perform quadriamputation was discussed after recovery from the critical period, when she became able to give her consent. Surgery was performed two months after the first signs of peripheral gangrene. Renal function recovered and the patient was further transferred for rehabilitation.

## 3. Discussion

Symmetrical peripheral gangrene (SPG) may be encountered after* Plasmodium falciparum* malaria but remains rare in travelers returning from endemic areas [1–13]. It is usually associated with the most severe forms complicated by multiple organ failure and is regarded as a consequence of distal vessel obstruction by fragments of parasites and erythrocytes and disseminated intravascular coagulation (DIC). Mortality rate may be as high as 35%, with a rate of amputation ranging from 70% to 90% [12]. In most of the published cases reporting on SPG, parasitemia is either not mentioned or not exceptionally elevated [13]. The effect of the treatment may not be underestimated and, in the present observation, the patient had already received a single loading dose of artemether-lumefantrine before the first determination of parasitemia. SPG appears usually within the first 3 days of effective antimalarial therapy, at which time parasitemia is often very significantly reduced [3]. As in our observation, SPG may develop before any bacterial infection. It appears therefore that the blood coagulation cascade is initially activated by changes in the erythrocyte membrane following* Plasmodium falciparum infection* and by the release of inflammatory cytokines leading to thrombin formation [5]. There is also a recent interest for the determination of ADAMTS13 activity in severe malaria. A deficiency in ADAMTS13 (a von Willebrand factor (VWF) cleaving protease) is associated with accumulation of prothrombogenic unusually large VWF multimers in plasma. It has been reported that symptomatic* Plasmodium falciparum infection* was associated with a significant increase in VWF and active VWF levels and a decrease in ADAMTS13 activity, resulting in the presence of circulating ultra-large VWF multimers [14]. The presence of reduced ADAMTS13 activity may contribute to the pathophysiological changes observed in severe malaria and particularly to microvascular disorders as observed in other groups of thrombotic microangiopathy. Till now, there is no direct link between ADAMTS13 activity and the occurrence of SPG.

The role of concomitant therapy in the pathogenesis of SPG has also to be discussed. In a review of 23 cases of* Plasmodium falciparum* malaria associated with DIC and SPG, 17 had received quinine as antimalarial therapy [3]. Patients treated with high doses of norepinephrine are also particularly at risk of developing SPG [7].

There is no evidence that blood exchange transfusions or fresh frozen plasma administration could be effective treatment options for patients with DIC and SPG. Heparin or streptokinase is not indicated. Surgery is often performed after some delay, when the delimitation of dry necrotic areas is evident.

Severely ill patients with complicated* Plasmodium falciparum* malaria are also profoundly immunosuppressed and susceptible to opportunistic fungal infections [15, 16]. Invasive candidiasis was reported in a single case of a nonimmune previously healthy 47-year-old man with* Plasmodium falciparum *malaria with a high parasite count (24%), shock, and multiple organ failure [15]. Blood cultures were positive for* Candida albicans* from admission. The patient had a favourable outcome after amphotericin B plus flucytosine therapy. Several factors may be involved in this acquired immunosuppression: free iron overload as a result of massive hemolysis, immunosuppressive effects of some antimalarial drugs, interference of the organism with innate cellular and specific cell-mediated immunity… [16]. Repeated candidemia and bacteraemia in the present observation may also be related to a breakdown in intestinal mucosal barrier caused by disturbances in the microcirculation [17, 18].

In conclusion, by some aspects, the clinical course of severe* Plasmodium falciparum *malaria may mimic the evolution of other thrombotic microangiopathies. The limitation of this observation is that some specific coagulation tests (VWF multimers, prothrombin fragment 1 + 2, and thrombin antithrombin complexes) could not be obtained.

Cytoadherence of parasitized erythrocytes causes not only mechanical obstruction but also endothelial activation. Further investigations of the coagulation cascade could be helpful to better investigate microcirculatory disorders and subsequent ischemic injuries.
